# A Convenient Truth: Cost of Medications Need Not Be a Barrier to Hepatitis B Treatment

**DOI:** 10.9745/GHSP-D-16-00128

**Published:** 2016-06-20

**Authors:** Matthew Barnhart

**Affiliations:** a Global Health, Science and Practice, Associate Editor, Washington, DC, USA

## Abstract

Drugs that are inexpensive to manufacture and simple to administer greatly expand the potential to help tens of millions of people who need treatment for chronic hepatitis B virus (HBV) infection. Key program implementation challenges include identifying who would benefit from antiviral medication and ensuring long-term and consistent treatment to people who feel well. The best opportunities are where health systems are advanced enough to effectively address these challenges and in settings where HIV service platforms can be leveraged. Research, innovation, and collaboration are critical to implement services most efficiently and to realize economies of scale to drive down costs of health care services, drugs, and diagnostics.

Viral hepatitis, principally due to chronic hepatitis B virus (HBV) and chronic hepatitis C virus (HCV), claimed 1.4 million lives worldwide in 2013,^1^ a rising toll that is now actually greater than that of mortality from HIV. Of the annual deaths caused by viral hepatitis, almost half (686,000) are attributable to HBV.^1^

The toll of viral hepatitis is now greater than that of mortality from HIV.

Although HBV vaccination rates for the childhood routine hepatitis B vaccine series were 82% globally in 2014,^2^ coverage rates for the hepatitis B birth dose—to optimally prevent mother-to-child (perinatal) transmission—lag behind. Furthermore, global disability-adjusted life years lost due to HBV-associated liver cancer have continued to rise by 4.8% since 2005.^3^ This is because the vast majority of complications from HBV occur among individuals older than 40 who were infected in the perinatal period or as young children. Indeed, an estimated 240 million individuals are already chronically infected,^4^ of whom 20% to 30% will eventually develop cirrhosis and/or liver cancer in the absence of treatment.^5^

Although chronic hepatitis B (CHB) infection is usually not curable, thankfully certain antiviral drugs are highly effective at suppressing viral replication and preventing the progress of liver damage without the development of resistance. A momentum is now building to expand access to these medications, in keeping with a new commitment within the Sustainable Development Goals to “combat hepatitis.”^6^ In 2015, the World Health Organization (WHO) issued its first-ever recommendations for prevention, care, and treatment for persons with CHB,^5^ and at the 2016 World Health Assembly a global health sector strategy on viral hepatitis was unanimously adopted^7^ that includes targets to treat 5 million people with CHB by 2020 and 80% of people in need by 2030.^8^ While not all 240 million persons living with CHB need treatment, the number who would benefit from medication is enormous—84 million people by one estimate.^9^

WHO-issued targets aim to reach 80% of people with chronic hepatitis B in need of treatment by 2030.

Considering that CHB treatment is lifelong in many cases, it might at first glance seem unaffordable to seek to expand treatment access to so many. But here is the convenient truth: CHB medications are simple to administer and potentially very inexpensive.

## LOW-COST HBV MEDICATIONS CAN CREATE NEW OPPORTUNITIES

Unlike treatment for HIV or tuberculosis, in the substantial majority of CHB cases a single antiviral agent with a high barrier to resistance can effectively suppress HBV. WHO recommendations provide 2 options for preferred first-line agents, tenofovir disoproxil fumarate (TDF) or entecavir (ETV),^5^ and rates of virologic resistance are vanishingly low with either when used in treatment-naïve patients: 0% with long-term TDF^10^ and 1.2% at 5 years with ETV.^11^ ETV and TDF both have a good safety and tolerability profile, although WHO recommends kidney function monitoring tests for people receiving either agent.^5^ Long-term TDF use has been associated with loss of bone mineral density, and ETV must be avoided in pregnant women due to evidence of harm in animal studies.

In most cases of chronic hepatitis B infection, a single antiviral agent with a high barrier to resistance can effectively suppress the virus.

TDF is already a backbone of HIV treatment at the same dosage approved for HBV treatment (300 mg/day) and had a ceiling price in 2015 of US$48 per patient year as a generic for HIV^12^; that price would be quite affordable for CHB treatment in middle-income countries, although less than ideal for low-income countries. On the other hand, ETV requires a very low dose of 0.5 mg/day (600-fold less than TDF), with an estimated cost of producing the active pharmaceutical ingredient (API) at scale of only US$2–$4 per year per a recent comprehensive analysis by Hill et al.,^13^ which is one-sixth to one-twelfth the per-pill cost of API compared with TDF. Using quite conservative estimates, this analysis arrives at a price estimate of US$36 per year for generic ETV. However, since the API cost per patient year for ETV should be about US$20 less than TDF, it would also be reasonable to estimate that at high volumes the price of generic ETV could differ from the price of TDF by around US$20. Thus, if WHO’s 2030 goal of reaching 80% of people in need of CHB with treatment—over 50 million people—were met, the potential cost savings in using generic ETV rather than TDF might be close to US$1 billion per year (although pregnant women and people living with HIV who were co-infected with HBV would still need to use TDF). ETV’s low dose may also lower in-country logistic costs for transport and storage at sites.

## PATENT EXPIRATIONS WILL HELP

While it is potentially very inexpensive to manufacture generic ETV, current prices of the drug are unfortunately very high, with a lowest global price of US$427 for a generic version not approved by a stringent regulatory authority such as the U.S. Food and Drug Administration, and US$6,127 for a generic version sold in the United States.^13^ This is because use of entecavir is currently very low, due in part to the very high prices of branded ETV and TDF, which have to some degree limited the uptake of these drugs in middle- and high-income countries (2015 originator prices in the United States were US$15,111 for ETV and US$10,718 for TDF).^13^ However, patents on ETV have recently expired in much of the world, including in the United States, and TDF’s main patents will have expired by 2018 in most countries. This will create a dramatically different situation, as it should enable patients throughout the world to receive the most effective treatments while also making it economically feasible to explore the provision of treatment earlier in infection using a simplified public health approach.

## PRIORITIZING RECIPIENTS

WHO’s guidance appropriately recommends that treatment be prioritized first for patients who have cirrhosis. To prevent HBV-associated liver cancer and cirrhosis, however, it will be crucial to treat people *before* they develop cirrhosis,^14^ since liver inflammation occurring over many years predisposes to cancer development and end-stage liver disease. To identify patients without cirrhosis who are at high long-term risk of liver cancer, WHO recommends primarily using quantitative HBV DNA (viral load, or VL) testing,^5^ the key biomarker shown to correlate most closely with future risk.^15^ However, as HBV DNA VL testing may not be available in the near term in many low-resource settings due to cost and implementation constraints, WHO recommendations also allow for treatment of individuals over age 30 who have persistent elevations of liver enzymes (a conditional recommendation with a low quality of evidence).^5^ More research is needed to characterize easy-to-assess CHB prognostic factors for the development of HBV-related cirrhosis and liver cancer, including the relative benefits of treatment in preventing liver cancer in different disease stages, epidemiologic settings, and populations. This includes in sub-Saharan Africa, a region of the world where there is a dearth of robust prospective data despite having high prevalence overall and extremely high prevalence in many countries—for example 22% in South Sudan and 14% in Zimbabwe.^4^

## KEY CHALLENGES: TESTING, TREATMENT ELIGIBILITY, AND RETENTION

Despite the low costs and clear benefits of antivirals, drugs will not be a magic bullet in and of themselves. To begin with, the proportion of people who have been tested for HBV is very low in most countries; WHO estimates that globally less than 5% of people living with chronic HBV and/or HCV are aware of their status.^8^ On a more positive note, even a one-time HBV test for adults could enable the identification of most people who would benefit from treatment, because the vast majority of HBV-associated liver cancer and cirrhosis occurs among people who were infected perinatally or as children. Rapid, point-of-care tests have been developed for HBV^16^ and could boost efficiencies. However, the majority of rapid tests have not yet been prequalified by WHO, and multiple tests lack international validation of sensitivity and specificity in the populations in which they might be used.

The proportion of people tested for HBV is very low in most countries, but rapid point-of-care tests could help.

Beyond testing, other substantial implementation challenges include linkage to longitudinal care services and differentiating between patients qualifying for treatment now versus those who only need regular monitoring. In addition to providing drugs for long-term daily intake, programs will also need to convince people who feel healthy to adhere to long-term medication. While this is a challenge common to many chronic diseases, it is very salient for HBV, since only a minority of people living with chronic infection will die from its consequences, which often occur several decades after diagnosis. Engaging and retaining men into long-term treatment is also critical for CHB treatment programs, particularly because men have a more than threefold higher risk for HBV-associated liver cancer than women (27% vs. 8% lifetime risk, respectively, among those infected in the perinatal period).^17^ Retention of patients on antivirals is important not only to reduce risk of liver cancer and cirrhosis but also to avoid hepatic “flares”—serious and sometimes life-threatening increases in liver inflammation that can occur for several reasons among people living with HBV, including due to an immune response to virus when it resurges after medications are stopped.^18^

## LEVERAGING HIV PLATFORMS

Scaling up any new service entails significant organizational effort and cost. In light of these various hurdles, the best opportunities to expand CHB treatment rapidly may occur where health systems are relatively advanced, for example, in middle-income countries in Asia that have a high HBV burden. However, significant programmatic synergies with HIV platforms also exist, which could help enable service delivery even in low-income countries with less robust health systems, including many in sub-Saharan Africa that have extensive HIV treatment programs. Supply chain, laboratory testing, and longitudinal care systems for HIV treatment have great commonality with HBV treatment. Even for more complex elements of HBV care such as viral load testing, promising opportunities exist to leverage existing HIV infrastructure, as easy-to-transport dried-blood spots that are used for HIV DNA-PCR testing can also be used to quantify HBV DNA^19^ and test Hepatitis B e antigen (HBeAg),^20^ 2 strong markers of risk for developing liver cancer. HBV treatment programs can also draw on lessons from community outreach, peer support, and service delivery models that HIV programs have developed, including approaches to increase male engagement and to support adherence and retention.

Significant programmatic synergies with HIV platforms exist, which could help enable hepatitis B treatment programs in countries with less advanced health systems.

## COLLABORATION, INNOVATION, AND RESEARCH

Most countries with high burdens of HBV have growing economies that can mobilize their own domestic resources to support the increased costs of expanding CHB treatment, which ultimately may save costs by reducing long-term medical expenses for liver cancer and cirrhosis. But to enable optimal access to generic ETV and the variety of HBV-related laboratory tests that will be needed, countries will benefit from actively working together to achieve greater economies of scale, using approaches such as coordinated ordering and prequalification of products to address regulatory bottlenecks. New technologies will also be important to drive down costs and improve outcomes. One such example is tenofovir alafenamide (TAF), a second-generation prodrug of tenofovir recently reported to be non-inferior to TDF in phase III trials of HBV treatment.^21^ TAF causes less bone and renal toxicity than TDF and should be less expensive, as it is effective at a low dose of 25 mg/day, although as a patent-protected drug it may be very expensive for many countries. It is also quite possible that the dose of TDF needed to treat HBV effectively may be markedly less than for HIV, as in *in vivo* animal studies TDF produces about 50% of the levels of the active metabolites in liver cells compared with TAF on a per mg basis.^22^ This suggests that dose-reduction studies of TDF for CHB treatment may be an alternate avenue to explore to reduce cost and toxicity. Low-dose agents such as TAF, and especially ETV, might also be amenable to long-acting implants,^23^ which, along with technology platforms such as mHealth, may improve long-term adherence. And research toward a cure for CHB remains important.^24^ Along with therapeutic advances, innovations in laboratory testing are needed, such as point-of-care liver function tests^25^ and easier-to-use viral DNA assays. Lastly, and perhaps most importantly, the global health community should learn by doing together, collaborating on a technical level to develop optimal delivery models for specific contexts and conducting joint research to provide better information about which patients would benefit from CHB treatment. Given the universally low CHB treatment access that currently exists in low-, middle-, and high-income countries, many people throughout the world would be helped from such technical collaborative efforts done in a true spirit of global health partnership.

To enable optimal access to low-cost HBV drugs and laboratory tests, countries will benefit from working together to achieve economies of scale.

## Notes

**First Published Online:** 2016 Jun 16
